# Traumatic Carotid Cavernous Fistula Resulting in Symptoms in the Ipsilateral Eye: A Case Report

**DOI:** 10.7759/cureus.30950

**Published:** 2022-10-31

**Authors:** Muhammad Salman Saleem, Sai Sreya Yadlapalli, Sidra Jamil, Favour C Mekowulu, Muhammad Saad, Ahmad Sadiq, Umair Rashid, Farhan Saleem

**Affiliations:** 1 Medicine, Lahore General Hospital, Lahore, PAK; 2 Internal Medicine, Spartan Health Sciences University, Vieux Fort, LCA; 3 Internal Medicine, California Institute of Behavioral Neurosciences and Psychology, Fairfield, USA; 4 Internal Medicine, V.N. Karazin Kharkiv National University, Kharkiv, UKR; 5 Interventional Neuroradiology, Lahore General Hospital, Lahore, PAK; 6 Orthopaedic Surgery, Lahore General Hospital, Lahore , PAK

**Keywords:** carotid, carotid cavernous fistula, traumatic carotid cavernous fistula, trauma, ccf

## Abstract

Post-traumatic unilateral carotid cavernous fistula (CCF) with ipsilateral symptoms is a rare occurrence, so its diagnosis frequently gets overlooked for other more common conditions. Timely imaging with digital subtraction angiography (DSA) and appropriate vascular intervention is essential in preventing potentially serious sequelae in such cases. We describe a case of post-traumatic CCF in a 42-year-old man who experienced intermittent headaches and right eye redness, proptosis, and watery discharge for three months following the incident. He was diagnosed with a right CCF based on DSA. Timely endovascular embolization with the coiling method resulted in obvious relief of the ocular symptoms and an improved prognosis. This article offers a description of our patient, a brief discussion of the existing literature, the challenges of diagnosing such cases, and a variety of therapy options.

## Introduction

Carotid cavernous fistula (CCF) is an abnormal vascular connection between the internal carotid artery (ICA) or the external carotid artery (ECA) and the venous branches of the cavernous sinus (CS); it is rare but can cause serious complications. Etiologically, CCF may be caused by head trauma most commonly, which may be blunt trauma to the brain or more notably, a basilar skull fracture resulting in a tear of the intracavernous ICA [1]. Other causes include the spontaneous rupture of a cavernous carotid aneurysm or a weakness of the arterial wall [2]. CCF is typically divided into direct and indirect types. Shunting of the blood from the carotid artery to the cavernous sinus increases the pressure inside the cavernous sinus, causes the backing up of blood in the draining vessels, may cause the flow to reverse, and presents clinical features that resemble many conditions of the eye and neck. Therefore, it is important for neurologists, neurosurgeons, and ophthalmologists to focus on the diagnosis of CCF.

## Case presentation

A 42-year-old man presented to our hospital with a complaint of redness in the right eye along with proptosis and watery discharge for three months. This was associated with intermittent headaches. The headache was gradual in onset with no change in severity. The headache was generalized with no diurnal rhythm and no prodromal symptoms. There were no aggravating factors, but the headache was partly relieved by analgesics. It was not associated with fever, neck stiffness, tinnitus, and projectile vomiting. There was no history of nausea, vomiting, blurring of vision, double vision, photophobia, irritation, tachycardia, heat intolerance, or tremors. A detailed neurological examination of the patient was unremarkable. The patient was well-orientated in time, place, and person. All of his cranial nerves were intact. The patient’s higher mental functions were intact, and he had a score of 29 on the mini-mental state examination (MMSE). His memory was preserved with no signs of amnesia. His eye examination was remarkable for redness, proptosis, and watery discharge in his right eye. No miosis (small pupil), enophthalmos, and loss of sweating on the face were noted, which ruled out Horner’s syndrome.

On inquiry, the patient revealed a history of a road traffic accident six months back in which he suffered traumatic injuries resulting in a fracture of the left fibula, lacerations on the forehead, and grazing of the thighs and hips. There was also bleeding in the ear, nose, and throat. A CT scan done after the accident did not reveal an intracranial bleed or any other cranial deformity. However, three months after the accident, he developed symptoms in the right eye.

The patient revealed no past medical history of diabetes, hypertension, tuberculosis, or hepatitis. No past surgical history. He took medication for asthma 10 years ago but was not on any such medication currently. He had been taking appetite stimulants and analgesics for headaches for the past three months and did not report allergies to any medications. He was a non-smoker but had been consuming naswar (smokeless tobacco) for the past 25 years.

**Figure 1:**
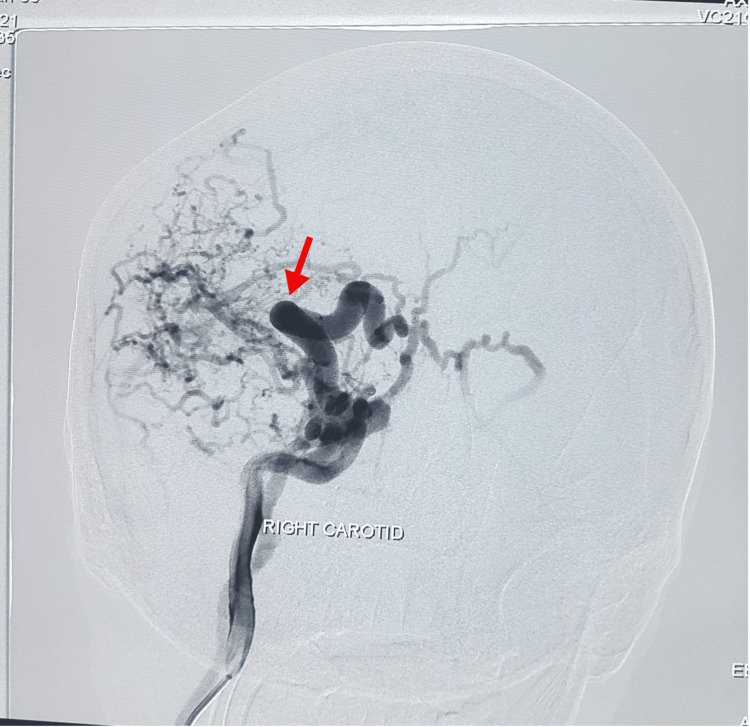
Digital subtraction angiography right carotid showing high flow carotid cavernous fistula fistula draining into cavernous sinus and adjacent cortical veins (pre-op)

A provisional diagnosis of CCF was made as digital subtraction angiography (DSA) revealed a right carotid-cavernous fistula, Figure 1.

**Figure 2:**
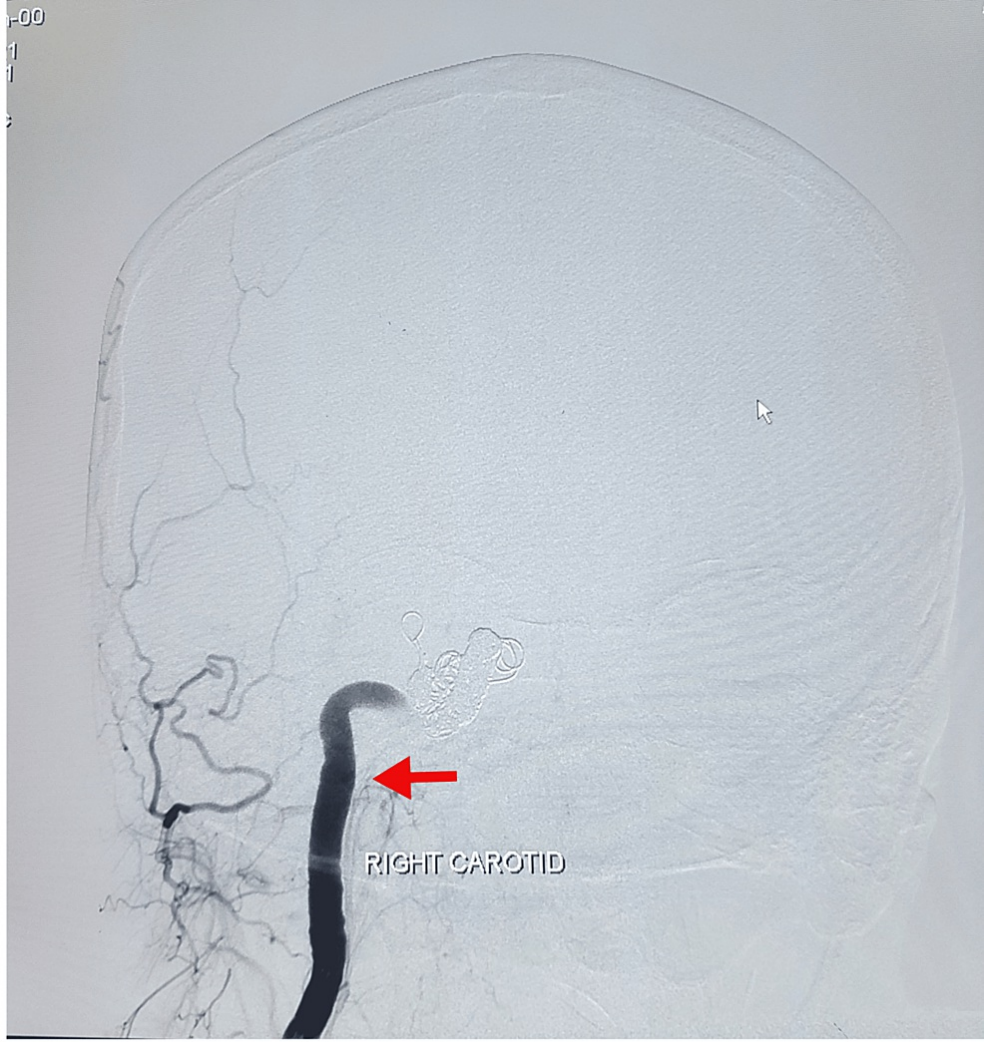
Coil placed in right carotid artery (post-op)

**Figure 3:**
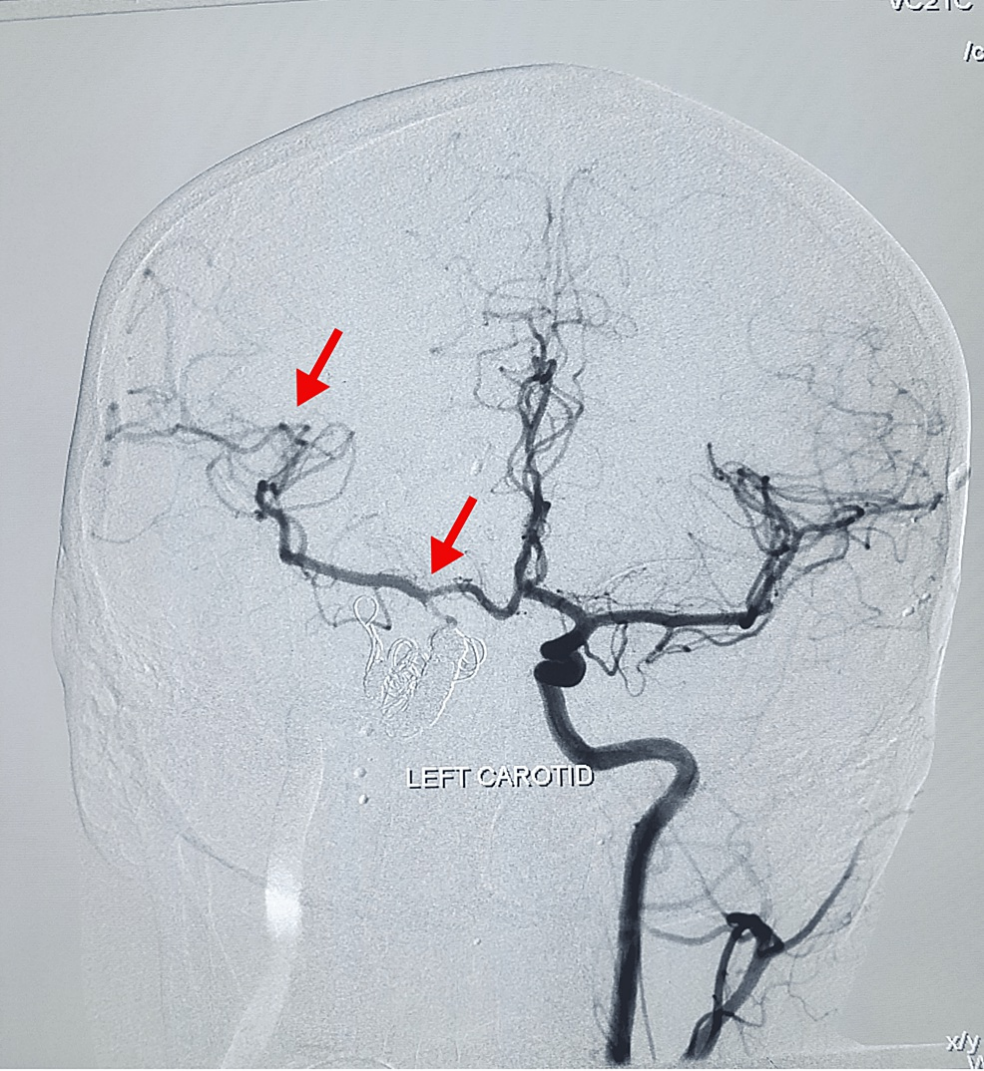
Digital subtraction angiography left carotid showing alternate blood supply through left anterior communicating branch to the right cerebrum

The endovascular embolization by coiling method was suggested by the interventional neuroradiology department. The procedure was performed under general anesthesia. The right femoral artery was engaged via the 6Fr sheath. Parent artery occlusion for the right CCF was done with metallic coils. Anterior communicating artery aneurysm filling from the right anterior cerebral artery was densely packed with coils. The ipsilateral hemisphere was getting supply from the contralateral internal carotid artery through the anterior communicating artery. No vasospasm was seen in post-procedural angiograms. Homeostasis was achieved through manual compression. The femoral sheath was intact, and the catheter was removed after 24 hours. The patient left the procedure room in stable condition. A post-operative DSA of the left carotid was performed, which showed alternate blood supply through the left anterior communicating branch to the right cerebrum (Figure 2), and the patient recovered well afterwards. A post-procedure DSA of the left carotid was done, which showed alternate blood supply through the left anterior communicating branch to the right cerebrum (Figure 3).

The patient was asked to follow up six days after the procedure, and almost complete relief in his ocular symptoms of proptosis, redness, and watery discharge from the right eye was noted. There was also improvement in his complaint of intermittent headache; however, he did report a few episodes of it, for which he was advised to continue taking analgesics. The patient was then asked to follow up after six weeks. On his return after six weeks, he reported the complete absence of any episodes of headache and was able to continue with his usual daily activities. The patient was asked to follow-up after every six months for further check-ups.

## Discussion

Etiology and classification

Carotid cavernous fistulas arise when an abnormal vascular connection forms between the CS and the ICA and/or ECA or their branches. Etiologically, CCF may be caused by head trauma most commonly, which may be blunt trauma to the brain or more notably, a basilar skull fracture resulting in a tear of the intracavernous ICA [1]. Trauma without fracture may cause distal compression of the ICA, leading to increased intraluminal pressure and proximal rupture into the CS [2]. Less commonly CCF may occur spontaneously from rupture of an aneurysm or a genetic disease predisposing to vessel wall weakness, e.g., Ehlers-Danlos Syndrome, fibromuscular dysplasia, and pseudoxanthoma elasticum, whereby minor increases in blood pressure result in rupture [3-6]. CCF may also be rarely caused by iatrogenic injury during craniotomy, carotid endarterectomy, transsphenoidal exploration, endovascular procedures, and sinus surgery [7-13].

CCF can be classified in multiple ways. Etiologically, it is classified into traumatic and spontaneous types; hemodynamically, into high-flow and low-flow types; and anatomically, into direct and indirect types, with direct CCF referring to a fistula between ICA/ECA and CS and indirect CCF referring to a fistula between branches of ICA/ECA and CS. Barrow et al. classified CCF into 4 types: A, B, C, and D. Type A arises from intracavernous ICA; type B from meningeal branches of ICA; type C from meningeal branches of ECA; and type D from meningeal branches of ICA and ECA both [7].

Pathophysiology

Venous drainage of the eye, namely the superior ophthalmic vein (SOV) and inferior ophthalmic vein (IOV), drains into the CS. Shunting of arterial blood into CS results in increased pressure in CS and backup of blood in the SOV and IOV, which causes the proptosis, chemosis, and other cardinal features seen in CCF patients. Congestion of the episcleral veins causes an increase in IOP and resultant secondary glaucoma [14-16]. The retinal artery perfusion pressure is also reduced, leading to retinal ischemia and visual disturbances. Dilation of CS due to increased blood volume may compress the structures inside and adjacent to CS, i.e., CN III, IV, V1, V2, VI, and lead to ophthalmoplegia [14].

Clinical presentation and differential diagnosis

CCF presents most commonly with proptosis, chemosis of the conjunctiva, orbital bruit, and headache. Most patients report visual disturbances such as diplopia and blurring of vision. Corkscrew episcleral vessels may also be prominent. Cranial nerve palsies may present with ophthalmoplegia, and cases with long-standing untreated proptosis may be complicated by exposure keratopathy [17]. Proptosis of CCF may be confused with Graves’ disease, which can be excluded by observing other systemic signs of hyperthyroidism. Conjunctivitis can be excluded by the presence of corkscrew hyperemia of the cornea and the absence of a history of infection. Primary glaucoma may also be suspected, but these patients do not respond to common anti-glaucoma therapies. Ophthalmoplegia may be confused with ocular myasthenia, which can be excluded by checking for acetylcholinesterase antibodies, neostigmine tests, and thymus CT [18].

Imaging examination

Most patients with CCF will undergo non-invasive imaging tests such as CT and MRI to aid in diagnosis. A CT scan is better at visualizing bone fractures, whereas an MRI can better delineate veins and edema. Features such as CS enlargement, orbital edema, extraocular muscle enlargement, dilation of SOV, and basilar skull fractures are readily evident on CT and MRI and are strongly suggestive of CCF [19,20]. However, sometimes these features may not be apparent on CT/MRI, and so the gold standard of diagnosis is DSA. DSA has the advantage of visualizing small blood vessels that may be missed on MRI and blood flow aberrations; thus, any fistula or filling defect is readily picked up. Furthermore, it shows the location of the fistula and the extent of arteriovenous shunting. So, DSA is essential for planning interventions and assessing the angioarchitecture of CCF. DSA, being an invasive procedure, does carry a risk of cerebral vasospasm, thrombosis, or hemorrhage, but it still remains the confirmatory investigation of choice [18]. In addition to imaging (CTA, MRA, and DSA), there are other tests that are routinely done in such cases, including tonometry, pneumotonometry, ultrasonography, and or color Doppler imaging depending upon the neurological involvement of the patient. However, in our case, these tests were not required and a definitive diagnosis was made based on DSA.

Treatment

Multiple approaches can be used for the treatment of CCF, such as conservative management, open surgery, i.e., ICA ligation, radiosurgery, and endovascular embolization. Some patients may even undergo spontaneous closure of the fistula [21]. Conservative management involves external manual compression of the ipsilateral cervical carotid artery 4-6 times a week, but it has questionable efficacy for direct, high-flow fistulas. ICA Ligation was performed historically for CCF, but this procedure had a high mortality rate, prompting the search for better treatment options. Nowadays, endovascular embolization is the hallmark of CCF treatment, which involves using a balloon catheter, metallic coil, or liquid embolic agents to occlude the fistula site [22]. Transarterial access is used for branches of ECA and certain direct fistulas, whereas transvenous access is employed for ICA fistulas.

In our case, consent was obtained from the patient. The procedure was performed under general anesthesia. The right femoral artery was engaged via the 6Fr sheath. Parent artery occlusion for the right CCF was done with metallic coils. Anterior communicating artery aneurysm filling from the right anterior cerebral artery was densely packed with coils. The ipsilateral hemisphere was getting supply from the contralateral internal carotid artery through the anterior communicating artery. No vasospasm was seen in post-procedural angiograms. Homeostasis was achieved through manual compression. The femoral sheath was intact, and the catheter was removed after 24 hours. The patient left the procedure room in stable condition.

## Conclusions

Most patients who are later diagnosed with a case of CCF initially usually present to an ophthalmologist, and hence, it is of paramount importance that an ophthalmologist can recognize this condition by its most common clinical presentation. If special consideration is given to the mode of injury (trauma), vessel, or hemodynamic diseases during history-taking and physical examination, then a prompt diagnosis can be made possible. It is evident that early clinical and radiological diagnosis of CCF and endovascular embolization leads to an excellent prognosis and outcome and can prevent potentially severe consequences. The final diagnosis of CCF must always, if possible, be confirmed using a DSA examination. This way, correct and efficient treatment can be ensured. Since these patients primarily present with ophthalmological concerns, ophthalmologists must remain on the lookout for a possible CCF if diagnosis eludes them.

## References

[1] Yoo K, Krisht AF (2000). The cavernous sinus: a comprehensive text. Etiology and Classification of Cavernous Carotid Fistulas.

[2] Helmke K, Krüger O, Laas R (1994). The direct carotid cavernous fistula: a clinical, pathoanatomical, and physical study. Acta Neurochir (Wien).

[3] Hirai T, Korogi Y, Goto K, Ogata N, Sakamoto Y, Takahashi M (1996). Carotid-cavernous sinus fistula and aneurysmal rupture associated with fibromuscular dysplasia. A case report. Acta Radiol.

[4] Kupersmith MJ, Berenstein A, Choi IS, Warren F, Flamm E (1988). Management of nontraumatic vascular shunts involving the cavernous sinus. Ophthalmology.

[5] Schievink WI, Piepgras DG, Earnest F 4th, Gordon H (1991). Spontaneous carotid-cavernous fistulae in Ehlers-Danlos syndrome type IV. Case report. J Neurosurg.

[6] Rios-Montenegro EN, Behrens MM, Hoyt WF (1972). Pseudoxanthoma elasticum. Association with bilateral carotid rete mirabile and unilateral carotid-cavernous sinus fistula. Arch Neurol.

[7] Barrow DL, Spector RH, Braun IF, Landman JA, Tindall SC, Tindall GT (1985). Classification and treatment of spontaneous carotid-cavernous sinus fistulas. J Neurosurg.

[8] Kupersmith MJ, Berenstein A, Flamm E, Ransohoff J (1986). Neuroophthalmologic abnormalities and intravascular therapy of traumatic carotid cavernous fistulas. Ophthalmology.

[9] Motarjeme A, Keifer JW (1973). Carotid-cavernous sinus fistula as a complication of carotid endarterectomy. A case report. Radiology.

[10] Pedersen RA, Troost BT, Schramm VL (1981). Carotid-cavernous sinus fistula after external ethmoid-sphenoid surgery. Clinical course and management. Arch Otolaryngol.

[11] Pigott TJ, Holland IM, Punt JA (1989). Carotico-cavernous fistula after trans-sphenoidal hypophysectomy. Br J Neurosurg.

[12] Sekhar LN, Heros RC, Kerber CW (1979). Carotid-cavernous fistula following percutaneous retrogasserian procedures. Report of two cases. J Neurosurg.

[13] Takahashi M, Killeffer F, Wilson G (1969). Iatrogenic carotid cavernous fistula. Case report. J Neurosurg.

[14] Fattahi TT, Brandt MT, Jenkins WS, Steinberg B (2003). Traumatic carotid-cavernous fistula: pathophysiology and treatment. J Craniofac Surg.

[15] Talks SJ, Salmon JF, Elston JS, Bron AJ (1997). Cavernous-dural fistula with secondary angle-closure glaucoma. Am J Ophthalmol.

[16] Ishijima K, Kashiwagi K, Nakano K, Shibuya T, Tsumura T, Tsukahara S (2003). Ocular manifestations and prognosis of secondary glaucoma in patients with carotid-cavernous fistula. Jpn J Ophthalmol.

[17] Chaudhry IA, Elkhamry SM, Al-Rashed W, Bosley TM (2009). Carotid cavernous fistula: ophthalmological implications. Middle East Afr J Ophthalmol.

[18] Zhu L, Liu B, Zhong J (2018). Post-traumatic right carotid-cavernous fistula resulting in symptoms in the contralateral eye: a case report and literature review. BMC Ophthalmol.

[19] Acierno MD, Trobe JD, Cornblath WT, Gebarski SS (1995). Painful oculomotor palsy caused by posterior-draining dural carotid cavernous fistulas. Arch Ophthalmol.

[20] de Keizer R (2003). Carotid-cavernous and orbital arteriovenous fistulas: ocular features, diagnostic and hemodynamic considerations in relation to visual impairment and morbidity. Orbit.

[21] Gemmete JJ, Chaudhary N, Pandey A, Ansari S (2010). Treatment of carotid cavernous fistulas. Curr Treat Options Neurol.

[22] Gupta AK, Purkayastha S, Krishnamoorthy T, Bodhey NK, Kapilamoorthy TR, Kesavadas C, Thomas B (2006). Endovascular treatment of direct carotid cavernous fistulae: a pictorial review. Neuroradiology.

